# The first non-prion pathogen identified: neurotropic influenza virus

**DOI:** 10.1080/19336896.2021.2015224

**Published:** 2022-01-03

**Authors:** Suehiro Sakaguchi, Hideyuki Hara

**Affiliations:** Division of Molecular Neurobiology, The Institute for Enzyme Research (KOSOKEN), Tokushima University, Tokushima 770-8503, Japan

**Keywords:** Prion, prion protein, prion disease, neurodegenerative disease, virus infection, conformational conversion, influenza virus, protein polymerization

## Abstract

The cellular isoform of prion protein, designated PrP^C^, is a membrane glycoprotein expressed most abundantly in the brain, particularly by neurons, and its conformational conversion into the abnormally folded, amyloidogenic isoform, PrP^Sc^, is an underlying mechanism in the pathogenesis of prion diseases, a group of neurodegenerative disorders in humans and animals. Most cases of these diseases are sporadic and their aetiologies are unknown. We recently found that a neurotropic strain of influenza A virus (IAV/WSN) caused the conversion of PrP^C^ into PrP^Sc^ and the subsequent formation of infectious prions in mouse neuroblastoma cells after infection. These results show that IAV/WSN is the first non-prion pathogen capable of inducing the conversion of PrP^C^ into PrP^Sc^ and propagating infectious prions in cultured neuronal cells, and also provide the intriguing possibility that IAV infection in neurons might be a cause of or be associated with sporadic prion diseases. Here, we present our findings of the IAV/WSN-induced conversion of PrP^C^ into PrP^Sc^ and subsequent propagation of infectious prions, and also discuss the biological significance of the conversion of PrP^C^ into PrP^Sc^ in virus infections.

## Introduction

Conformational conversion of the cellular isoform of prion protein, designated PrP^C^, into its abnormally folded, amyloidogenic isoform PrP^Sc^, is a key pathogenic event in prion diseases, or transmissible spongiform encephalopathies, a group of fatal neurodegenerative disorders including Creutzfeldt-Jakob disease (CJD) in humans and scrapie and bovine spongiform encephalopathy (BSE) in animals [1–3]. PrP^Sc^ is a β-sheet-rich molecule, with propensity to easily aggregate to form fibrils, and is relatively protease-resistant and detergent insoluble [4]. PrP^C^ is a membrane glycoprotein anchored to the plasma membrane via a glycosylphosphatidylinositol moiety and expressed most abundantly in the brain, particularly by neurons, and to lesser extents in other various non-neuronal tissues [5–7]. PrP^C^ is detergent-soluble and sensitive to protease digestion and structurally consists of two domains, the flexible non-structural N-terminal domain and the globular C-terminal domain with two short β-sheets and three α-helices [6,7]. Structural transition from α-helices to β-sheets has been proposed to be an underlying mechanism of the conformational conversion of PrP^C^ into PrP^Sc^ [4].

Prion diseases in humans manifest as sporadic, hereditary, and acquired disorders [8]. The most common human prion disease, accounting for 85–90% of the total cases, is sporadic CJD (sCJD) [9–11]. The aetiologies of sCJD remain unknown. 10–15% of cases belong to hereditary prion diseases, such as familial CJD, Gerstmann-Sträussler-Scheinker syndrome, and fatal familial insomnia [12,13]. These diseases are causatively linked to specific mutations in the PrP gene (*Prnp*) [12,13]. It has been postulated that mutated PrP molecules are structurally unstable, thereby going through conformational changes to form a PrP^Sc^ structure. The remaining cases, accounting for less than 1%, are those of acquired prion diseases, which include iatrogenic CJD (iCJD), variant CJD (vCJD), and kuru [14,15]. These diseases are caused by intra- or inter-species transmission of proteinaceous infectious particles, termed ‘prions’, which are believed to mainly consist, if not entirely, of PrP^Sc^ molecules. PrP^Sc^ molecules assemble to form an oligomeric structure, which has been considered to be the molecular nature of a prion, functioning as a seed or scaffold to recruit PrP^C^ and forcing its conformational conversion into PrP^Sc^ through a seeded polymerization mechanism [16,17]. iCJD is a disease caused by human-to-human transmission of prions via medical treatments or procedures [14]. vCJD is believed to be caused by transmission of prions from BSE [14]. Kuru is a disease spread among Fore people via ritualistic cannibalism in Papua New Guinea [15].

Many recent lines of research have suggested that virus infections might be a risk factor for many neurodegenerative diseases, including Alzheimer’s disease and Parkinson’s disease [18–22]. It has been also reported that virus glycoproteins, including SARS-CoV-2 spike S glycoprotein, could enhance intercellular spreading of the pathogenic protein aggregates in exosomes through interaction with their cognate cellular receptors [23], further demonstrating the relevance of virus infections to the pathogenesis of protein aggregate-associated neurodegenerative disorders. We have recently found that infection with neurotrophic influenza A virus (IAV) induced the conformational conversion of PrP^C^ into PrP^Sc^ and the subsequent formation of infectious prions in cultured neuronal cells [24]. These results show that neurotropic IAV is the first non-prion pathogen that is able to induce the conversion of PrP^C^ into PrP^Sc^ and propagate infectious prions in cultured cells, raising the intriguing possibility that IAV infection in neurons might be a cause of or be pathogenetically associated with sporadic prion diseases, including sCJD. Here, we introduce our current findings of the neurotropic IAV-triggered conversion of PrP^C^ into PrP^Sc^ and formation of infectious prions, and discuss its biological significance in virus infections.

## Neurotropic IAV infection in PrP conversion and prion propagation

Here, we pose the hypothesis that certain virus infections might affect the conformation of PrP^C^, thereby structurally destabilizing PrP^C^ to undergo conformational conversion into PrP^Sc^. Mouse neuroblastoma N2a cells are widely used for prion infection experiments because they are highly susceptible to various prions, provoking the conformational conversion of PrP^C^ into PrP^Sc^ as well as propagating prions after prion infection [25,26]. The prion susceptibility of N2a cells is markedly enhanced by overexpressing PrP^C^ in the cells [26]. We previously established a N2a cell line, termed N2aC24, which expresses transduced mouse PrP^C^ at high levels and showed that N2aC24 cells were highly susceptible to RML and 22 L scrapie prions, producing large amounts of PrP^Sc^ after prion infection [27]. Therefore, to explore our hypothesis, we infected N2aC24 cells with a neurotropic strain of influenza A/WSN/33 (H1N1) virus (hereafter referred to as IAV/WSN) [24]. We found that N2aC24 cells were highly susceptible to IAV/WSN infection, undergoing massive cell death after infection [24]. To our surprise, proteinase K (PK)-resistant fragments of PrP were detectable in N2aC24 cells infected with IAV/WSN at a low multiplicity of infection of 0.01 at 7 and 8 days post-infection on Western blotting with large amounts of total proteins (300 μg proteins) from the cell lysate [24]. Immunofluorescent staining of the cells with 132 anti-PrP monoclonal antibody, which has been demonstrated to specifically recognize PrP^Sc^ in prion-infected cells under partially denaturing conditions, also showed positive signals [24]. These results indicate that IAV/WSN infection could destabilize the protein structure of PrP^C^, then triggering its conformational conversion into PrP^Sc^ in N2aC24 cells.

We found that a small portion of IAV/WSN-infected N2aC24 cells survived the infection and grew continuously. We termed the surviving cells as N2aC24R1 cells and passaged them at a 1:10 ratio. The PK-resistant PrP fragments became clearly detectable in N2aC24R1 cells on Western blotting even with lower amounts of total proteins (30 μg proteins) of the cell lysates at passage 1, and they were increased up to passage 10 [24], indicating that the PK-resistant PrP molecules are propagating in N2aC24R1 cells. In contrast, no PK-resistant PrP was observed in N2aC24 cells even after IAV/WSN infection when IAV/WSN infection was blocked by anti-IAV/WSN mouse antisera or the anti-influenza agent oseltamivir [24]. These results not only confirm that IAV/WSN infection is essential for inducing the conversion of PrP^C^ into PrP^Sc^ in N2aC24 cells, but also rule out the possibility of the contamination of laboratory prions in the cell culture. We also showed that intracerebral inoculation with cell lysates from N2aC24R1 cells into mice caused prion disease, with PrP^Sc^ accumulation and spongiosis degeneration in their brains [24]. Taken together, these results indicate that IAV/WSN infection could induce the conformational conversion of PrP^C^ into PrP^Sc^ and the subsequent formation of infectious prions in N2aC24 cells.

## Possible mechanism for IAV/WSN-induced PrP conversion

**Figure 1.**
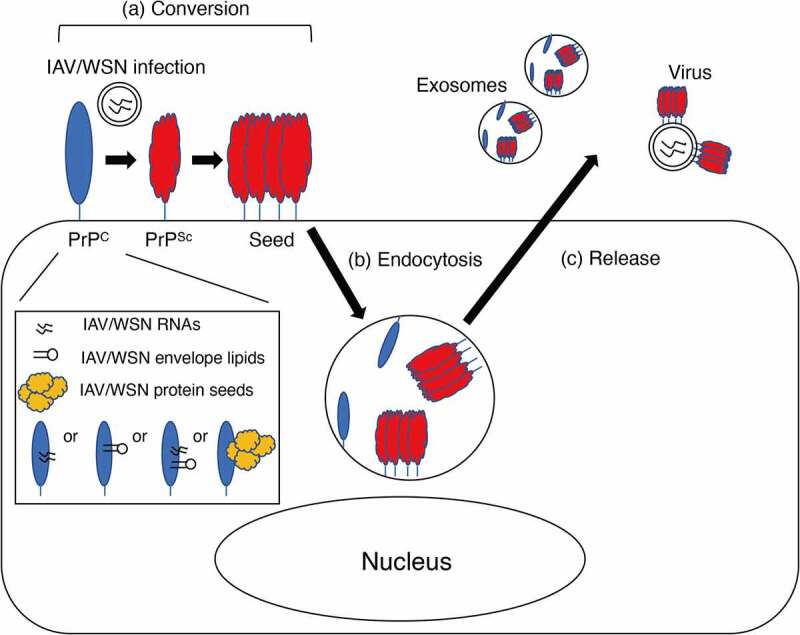
Possible effects of virus infections on various steps of PrP^Sc^ propagation. (a) IAV/WSN infection induces the conversion of PrP^C^ into PrP^Sc^, subsequently forming PrP^Sc^ seeds. IAV/WSN-derived RNA or lipid molecules or the protein seeds of IAV/WSN-derived proteins, such as PB1, PB2, and neuraminidase, might bind to and convert PrP^C^ into PrP^Sc^. (b) Infection with murine parvovirus stimulates intracellular internalization of PrP^Sc^ seeds in mouse A9 fibroblasts. (c) Molony murine leukaemia virus facilitates the incorporation of PrP^Sc^ into virus particles or exosomes, thereby increasing the release of PrP^Sc^ from 22L scrapie prion-infected NIH3T3 cells. Vesicular stomatitis virus glycoprotein and SARS-CoV-2 spike S might enhance intercellular spreading of exosomal PrP^Sc^ through interaction with their cognate cellular receptors.

We showed that IAV/WSN infection induced the conversion of PrP^C^ into PrP^Sc^ in parental N2a cells, which express only endogenous mouse PrP^C^ [24], indicating that overexpression of PrP^C^ is dispensable for IAV/WSN infection to induce the conversion of PrP^C^ into PrP^Sc^. Interestingly, IAV/WSN proteins examined, including HA, NS and M2, were no longer detectable in N2aC24R1 cells [24], indicating that persistent IAV/WSN infection is not necessary to maintain the conversion of PrP^C^ into PrP^Sc^ in N2aC24R1 cells. It is thus conceivable that PrP^Sc^ molecules nascently converted from PrP^C^ in N2aC24 cells after IAV/WSN infection might form oligomeric aggregates, or PrP^Sc^ seeds, subsequently recruiting other PrP^C^ molecules to them to convert them into PrP^Sc^ without the help of IAV/WSN and thereby enabling the constitutive conversion of PrP^C^ into PrP^Sc^ in N2aC24R1 cells even after IAV/WSN infection is cleared (Figure 1(a)). IAVs are negative-stranded, segmented, enveloped RNA viruses [28]. RNA and lipid molecules have been shown to bind to recombinant PrP and convert it into PK-resistant PrP [29,30]. Furthermore, *in vitro* PrP^Sc^ amplification techniques such as protein misfolding cyclic amplification (PMCA) or quaking-induced conversion techniques have shown that RNA and lipid molecules function as a cofactor for the conversion of PrP^C^ into PrP^Sc^ and the propagation of infectious prions [31]. It is thus possible that IAV/WSN-derived RNA or lipid molecules might play a role as a cofactor in the conversion of PrP^C^ into PrP^Sc^ in IAV/WSN-infected N2aC24 cells (Figure 1(a)). However, it was reported that RNA molecules from invertebrate species including bacteria, yeast, worms, and flies failed to convert PrP^C^ into PrP^Sc^ in PMCA [32]. It is thus interesting to investigate if IAV/WSN-derived RNA molecules might be able to function as a cofactor for the conversion of PrP^C^ into PrP^Sc^ in PMCA. Protein sequence analysis of eukaryotic viruses have identified many prion-like domains in various viral proteins [33]. Among IAV proteins, RNA-directed RNA polymerase catalytic subunit (PB1), polymerase basic protein 2 (PB2), and neuraminidase were shown to contain prion-like domains [33]. It is thus interesting to speculate that the prion-like domains of these IAV proteins might function as a seed for PrP^C^ to undergo conformational conversion into PrP^Sc^ (Figure 1(a)).

## Other virus infections in PrP conversion

Several reports have shown that virus infections could affect the inter- and intra-cellular dynamics of PrP^Sc^ in prion-infected cells. Infection with murine minute DNA virus, a murine parvovirus, in mouse A9 fibroblasts was shown to enhance the binding of exogenously added PrP^Sc^ molecules on the cell surface and stimulate their intracellular internalization (Figure 1(b)) [34]. Molony murine leukemia virus was also reported to facilitate the incorporation of PrP^Sc^ into virus particles or exosomes, thereby increasing the release of PrP^Sc^ into the culture medium of 22L scrapie prion-infected NIH3T3 cells (Figure 1(c)) [35]. It is thus possible that virus infections might increase the conversion rate of PrP^C^ into PrP^Sc^, by increasing the accessibility of PrP^C^ to PrP^Sc^ through enhancing the cell surface binding of PrP^Sc^ molecules and stimulating their internalization to lysosomal compartments, where PrP^Sc^ is supposed to convert PrP^C^ into PrP^Sc^, and/or increasing the release of PrP^Sc^ from prion-infected cells to prion-uninfected neighboring cells (Figure 1). Indeed, co-infection with scrapie prions and mouse adenovirus was reported to accelerate prion disease in mice, compared to infection with scrapie prions alone [36]. The Cork strain of caprine arthritis encephalitis virus, a small-ruminant lentivirus, was also shown to increase PrP^Sc^ levels in scrapie prion-infected primary sheep microglia [37]. In mice developing prion disease after intracerebral inoculation with ME7 prions, microglia highly responded to intranasal infection with Piry arbovirus, markedly proliferating in their brains compared to those in control mice infected with Piry arbovirus alone [38]. However, no data were available as to whether or not Piry arbovirus infection increased PrP^Sc^ levels in the brains [38]. Interestingly, vesicular stomatitis virus glycoprotein and SARS-CoV-2 spike S were shown to enhance intercellular spreading of protein aggregates, including those of Tau and PrP^Sc^, in exosomes into neighboring cells through interaction of the virus ligands with their cognate cellular receptors [23]. It is thus possible that IAV/WSN glycoprotein, or HA, might also be able to stimulate intercellular spreading of the nascently generated PrP^Sc^ in IAV/WSN-infected cells through a similar mechanism. However, whether or not these virus infections except for IAV/WSN could cause the *de novo* conversion of PrP^C^ into PrP^Sc^ remains unknown.

## Are prions anti-virus proteins?

Prion-like self-templating aggregates have been identified for other cellular proteins in mammals and non-mammals such as yeast, and suggested to be involved in certain cellular functions. The yeast protein Sup35, a yeast translation terminator protein, forms prion-like aggregates, termed [*PSI^+^*] prions [39]. It was indicated that [*PSI^+^*] prions could confer the yeast resistance to environmental changes through disturbing the translational termination of cellular proteins by impairing the normal function of Sup35 [40,41]. Mod5 also forms prion-like aggregates in yeast, thereby regulating the sterol biosynthetic pathway and eventually protecting the yeast from anti-fungal agents [42]. In mammals, prion-like protein polymerization has been identified for an innate immunity-associated protein, termed apoptosis-associated speck-like protein containing a caspase activation and recruitment domain (ASC) [43]. After virus infection, immune cells provoke prion-like polymerization of ASC to form the inflammatory signalling platform called inflammasome to elicit innate immunity against virus infection [43]. It is thus possible that prion-like polymerization of certain cellular proteins could be a cellular mechanism transducing defence signals against environmental stress including virus infection. Interestingly, the transgenic model mice of familial Alzheimer’s disease, which develop Aβ amyloid plaques in the brains from two months of age, were reported to be more resistant to intracerebral infection with human herpes virus-1 (HHV-1), showing lower mortality than control mice after infection [44]. These results suggest that Aβ peptides might function to be protective against virus infection, and that Aβ peptides might be produced as a cellular protective mechanism against virus infection. We previously showed that PrP^C^ could provide protection against infection with IAVs, including IAV/WSN, by demonstrating that mice devoid of PrP^C^ were highly vulnerable to IAV infections, succumbing to IAV-induced pneumonia with higher mortality than control mice [45]. For understanding of the biological significances of the conversion of PrP^C^ into PrP^Sc^ and the subsequent formation of infectious prions, it would be interesting to investigate whether the PrP conversion and the prion formation are associated with the cellular protective mechanism against virus infections.

## Conclusion

Emerging lines of evidence suggest that virus infections are a risk factor for many neurodegenerative disorders including Parkinson’s disease and Alzheimer’s disease [18–22]. Parkinson’s disease and Alzheimer’s disease are caused by accumulation of prion-like protein aggregates in the brain of disease-specific proteins, α-synuclein and Aβ, respectively [46–49]. It was shown that infection of neurotropic IAV (H1N1) caused aggregation of α-synuclein in neuron-like Lund human mesencephalic cells and in olfactory bulb neurons of mice [50]. Also, infection with the highly pathogenic, neurotropic H5N1 avian IAV has been shown to induce accumulation of phosphorylated α-synuclein in the substantia nigra pars compacta neurons in mice [20]. HHV-1 and −2, cytomegalovirus, and mumps virus have been also suggested to be associated with the pathogenesis of Parkinson’s disease [18,19]. HHV-1 infection was reported to increase Aβ amyloid peptides in human neuroblastoma and glioblastoma cultured cells as well as in the brains of mice [51]. We have shown that IAV/WSN infection induced the conversion of PrP^C^ into PrP^Sc^ and the subsequent formation of infectious prions in neuronal cultures cells [24], further highlighting the causative roles of virus infections in neurodegenerative disorders, including prion diseases.
